# Comparing the effectiveness of airway management devices in pre-hospital emergency care: A randomized clinical trial

**DOI:** 10.12669/pjms.314.7296

**Published:** 2015

**Authors:** Shahla Khosravan, Ali Alami, Arash Hamzei, Jalal Borna

**Affiliations:** 1 Shahla Khosravan, Msc, PhD in Nursing. Associate Professor of Nursing and Midwifery School, Social Determinants of Health Research Centre, Gonabad university of Medical Sciences, Gonabad, Iran; 2 Ali Alami, MD, PhD in Epidemiology. Assistant Professor of Health School, Social Determinants of Health Research Centre, Gonabad university of Medical Sciences, Gonabad, Iran; 3 Arash Hamzei, MD. Specialist in Anesthesiology, Assistant Professor of Faculty of Paramedics, Operating and Anaesthesia Department, Gonabad university of Medical Sciences, Gonabad, Iran; 4 Jalal Borna, Msc Medical-Surgical. Student Research Committee, Nursing and Midwifery School, Gonabad university of Medical Sciences, Gonabad, Iran

**Keywords:** Airway management, Endotracheal tube, Laryngeal mask airway, Oropharyngeal airway, Pre-hospital

## Abstract

**Objective:**

To assess the effectiveness of laryngeal mask airway, endotracheal tube and oropharyngeal airway for airway management in prehospital emergency care.

**Methods:**

The study sample of this randomized clinical trial was 54 patients needing pre-hospital airway management. All cases of intubation (ETI); after two failed attempts (37 patients), were randomly assigned to the oropharyngeal airway (OPA), and the laryngeal mask airway (LMA) groups. Patients’ hemodynamic, SaO2 and airway management parameters, were compared in three groups. The study data were analyzed by the Chi-square and one-way ANOVA, Bonferroni post-hoc, using SPSS, v. 18.0.

**Results:**

The results demonstrated that before and after the study, there was no significant difference among the study groups in terms of hemodynamic variables (P > 0.05) expect SaO_2_ (P < 0.001). The results also revealed that in the ETI group (n=17), the number of attempts and the time spent on inserting the airway device was significantly more than other two groups (P < 0.05).

**Conclusion:**

Laryngeal mask airway is as effective as oropharyngial airway for pre-hospital airway management by paramedics.

## INTRODUCTION

Airway obstruction—and subsequent oxygen deprivation—is an immediate threat to life and a real emergency.^1^,^2^ Consequently, Pre-hospital paramedics should be well qualified and prepared to manage patients airway with inadequate ventilation.^3^

Endotracheal intubation (ETI) is the gold standard for maintaining a patient airway.^4^ However, studies have revealed that compared with ETI in operative rooms and skill labs, pre-hospital intubation is a difficult and complicated task;^5^ especially less qualified healthcare providers have more problems with ETI,^6^,^7^ and compared with emergency physicians, their rate of unsuccessful intubation is greater.^6^ The European Resuscitation Council Guidelines for Resuscitation (2005) highlighted that only the qualified healthcare providers can perform ETI.^8^ Insertion of oropharyngeal airway (OPA) is a basic airway management technique that is widely employed by pre-hospital staff.^9^ Using OPA, patients maybe receive lower concentrations of oxygen and lower tidal volume.^10^

Supraglottic airway devices such as laryngeal mask airway (LMA) are alternative for airway management,^11^ widely used by anesthesiologists.^12^ In pre-hospital, the effectiveness of LMA in maintaining a patient airway and improving pulmonary ventilation as well as its complications, in the hands of paramedics, has remained less known; therefore we conducted this study aiming at comparing the effectiveness of three airway management devices including ETI, OPA, and LMA in real situation by paramedics.

## METHODS

The sample of this randomized clinical trial consisted of 54 patients needing pre-hospital airway management. Besides considering any contraindications for LMA insertion, other inclusion criteria for patients were having a Glasgow Coma Score (GCS) of less than nine, an age of more than eighteen, being hemodynamic parameters responses before and after intervention, being non-pregnant, no mouth injury, and a diagnosis of sever hypoxia or respiratory distress.

Endotracheal intubation is also the gold standard for maintaining a patient airway in Iran; therefore to consider this item, all the eligible patients (54 cases) were primarily subjected to ETI. In case of intubation failure after two attempts (37 cases), the patients were assigned to the OPA group (17 cases) or the LMA group (18 cases) by randomly allocation using Balanced Block Randomization. The inserted airway management device (OPA or Work™ LMA, size 4) was then connected to a manual resuscitation bag already connected to a portable oxygen delivery device. Patients received oxygen therapy and basic life support interventions until arriving at the accident and emergency department.

The primary endpoint measures were patients’ hemodynamic parameters including diastolic and systolic blood pressures (DBP and SBP), heart rate (HR), and the percentage of oxygen saturation (SaO_2_), were record at two time-points including before the beginning of airway management interventions and once arriving at the accident and emergency department. Moreover, we measured airway management parameters, the number of attempts and the time spent on inserting the intended airway device, the need to perform laryngoscopy and head positioning for facilitating the insertion of the device, airway management-related complications.

The study data were analyzed by using SPSS, v. 18.0. We employed the Chi-square, the one-way Analysis of Variance (one-way ANOVA), and Bonferroni post-hoc tests for data analysis. The level of significance was set at below 0.05.

The Ethics Committee of Gonabad University of Medical Sciences, Gonabad, Iran approved the study. Moreover, this trial is registered with IRCT registry ID: IRCT2012092310910N1. As the study participants were unconscious at the time of recruitment, we explained the aim and the process of the study to their family members and asked them to read and to sign the study informed consent form if they were accepted to participate in this study.

## RESULTS

Age, gender, and medical diagnosis of study groups are shown in Table-I. The study groups did not differ significantly in terms of age and medical diagnosis (Table-I), volume of serum and oxygen intake, distance and ambulance time of arrival to the emergency department (P > 0.05).

**Table-I:** Comparing the study groups in terms of age, gender, and medical diagnosis.

Patients’ characteristics	Study groups	OPA	LMA	ETI	P value
		N (%) / Mean ± SD	N (%) / Mean ± SD	N (%) / Mean ± SD
Sex	Male	15 (78.9)	15 (83.3)	7 (41.2)	0.013^†^
	Female	4 (21.1)	3 (16.7)	10 (58.8)
Age		48.53(23.0)	48.67(17.4)	60.71(21.0)	0.145^†^
Diagnosis	Trauma	11 (57.9)	10 (55.6)	7 (41.2)	0.562^†^
	Medical problems	8 (42.1)	8 (44.4)	10 (58.8)

† : The results of the Chi-square test.

The results demonstrated that before and after the study, there was no significant difference among the study groups in terms of DBP, SBP, HR, and SaO2 (P > 0.05; Table-II). However, there was a significant difference among the groups in terms of SaO2 after intervention (P < 0.001). The results of the Bonferroni post-hoc test showed that this difference was between the LMA and OPA groups (P value = 0.038) and between the ETI and the OPA groups (P value < 0.001). The results of the one-way ANOVA test and the Bonferroni post-hoc test also revealed that in the ETI group, the number of attempts for and the time spent on inserting the airway device was significantly more than the LMA and the OPA groups (P value < 0.05). However, the difference between the LMA and the OPA groups in terms of these two variables was not statistically significant (P value > 0.05; Table-III).

**Table-II:** Hemodynamic parameters in the study groups before and after the study.

Time	Before the study				After the study			
Group Variables	OPA	LMA	ETI	P value	OPA	LMA	ETI	P value*
SDP (mmHg)	117.2±38.8	119.5±41.1	128.0±31.0	0.683	113.2±24.4	113.8±26.4	117.5±43.5	0.916
DBP (mmHg)	75.3±25.1	72.8±20.8	74±23.7	0.955	74.1±15.3	71.0±12.1	70.9±24.1	0.834
HR (bit/min)	94.0±29.2	100.0±34.2	85.7±36.5	0.474	97.0±28.0	91.2±22.5	83.3±19.4	0.266
SaO_2_ (%)	61.3±16.8	71.0±16.5	70.2±19.0	0.204	84.7±11.6	92.0±6.85	96.8±1.96	0.000

* The results of the one-way ANOVA test.

**Table-III:** The airway management parameters in the study groups.

Patients’ characteristics	Study groups	OPA	LMA	ETI	P value
		N (%) / Mean ± SD	N (%) / Mean ± SD	N (%) / Mean ± SD
Sex	Male	15 (78.9)	15 (83.3)	7 (41.2)	0.013^†^
Time spent		1.16±0.37	1.06±0.23	1.94±0.96	<0.001*
Number of attempts		1.26±0.56	1.11±0.47	2.0±0.70	<0.001*
Head positioning	Yes	4 (21.1)	3 (16.7)	14 (82.4)	<0.001^†^
	No	15 (78.9)	15 (83.3)	3 (17.6)

* The results of the one-way ANOVA test.

† The results of the Chi-square test.

The results of this test also revealed that the number of patients in the ETI group who needed head positioning for facilitating the insertion of the device was significantly higher than the LMA and OPA groups (P < 0.001). Finally, none of the study participants developed airway management-related complications such as aspiration and regurgitation.

## DISCUSSION

The study findings revealed that the three groups did not differ significantly in terms of hemodynamic variables in the second monitoring (at emergency department). We did not find study which compares LMA with OPA; however while comparing facemasks with endotracheal intubation, supraglottic devices is considered less invasive and less interfering in hemodynamic response.^13^ This differences may be related to intervention situation (pre-hospital airway management in patients with severe hypoxemia). However, we found that after the study, the levels of SaO_2_ in both the LMA and ETI groups were significantly higher than the OPA group. This finding implies that LMA is more effective than OPA in improving SaO_2_ levels, and also as much effective as ETI which is the gold standard for maintaining a patient airway. Previous studies also indicated that LMA is as much effective as ETI in maintaining a patient airway.^1^,^11^,^14^ The distal end of an OPA opens at the pharynx, consequently, oxygen leakage is inevitable. Some researchers also reported a severe hypoxia secondary to air leakage from OPA. However, the distal end of endotracheal tube enters the trachea and the inflating cuff prevents air leakage. Similarly, the distal end of LMA completely covers the supraglottic area and opens directly at the trachea opening providing a direct airway passage to the trachea. The inflating cuff of LMA also minimizes air leakage. Moreover, the tongue-shaped pointed tip of LMA enters and obstructs esophagus, which prevents air from entering the esophagus and minimizes the risk of aspiration.^10^,^15^

The study findings also revealed that the number of attempts and the time spent on inserting the airway device in the ETI group were significantly more than the other two groups. Other studies^16^,^17^ have also reported a high success rate of over 80% for inserting LMA at the first attempt. On the other hand, Brimacombe et al. found that compared with the OPA, the number of attempts and the time spent on inserting LMA were significantly lower.^15^

Like other studies, we also observed that compared with the LMA and OPA groups, more patients in the ETI group needed head positioning for inserting the airway device.^10^,^18^ These findings demonstrate the supremacy of using LMA for airway management particularly in patients with spinal cord injuries who are at great risk for developing paraplegia.^19^

The study findings revealed that none of our patients developed complications such as regurgitation and aspiration. In other words, the study groups did not differ significantly in terms of airway management-related complications. Other study also found that compared with facemask, LMA was associated with fewer complications.^20^

## CONCLUSION

This study has been designed as a comparative effectiveness research in routine clinical practice that is different from a regular controlled clinical trial (such as manikin study^21^) for identifying evidence-based outcomes. The study findings are according to the paramedics self- report, and it seems further studies are needed. This study suggest that LMA is a simple, effective, and safe device for pre-hospital airway management.
